# Risk Governance in Clinical Education for Healthcare Students: A Scoping Review

**DOI:** 10.1111/tct.70355

**Published:** 2026-02-03

**Authors:** Raelynn R. Tong, Je Min Suh, Lisa Cheshire, Lucio Naccarella

**Affiliations:** ^1^ Melbourne Medical School The University of Melbourne Melbourne Australia; ^2^ Faculty of Medicine, Dentistry and Health Sciences The University of Melbourne Melbourne Australia; ^3^ Melbourne School of Population and Global Health The University of Melbourne Melbourne Australia

**Keywords:** clinical education, healthcare students, incident reporting, risk management

## Abstract

**Background:**

Clinical education is essential for the training and accreditation of healthcare students. It facilitates hands‐on application and prepares students for their future roles in a dynamic and complex workforce. However, such clinical exposure comes with inherent risk, not least due to the delicate balance between patient safety and student learning. Understanding risk is particularly crucial now as we confront new technologies that will undoubtedly transform clinical education. This scoping review aims to map current practices of tertiary institutions in managing risks and incidents in healthcare students' clinical education.

**Methods:**

This scoping review adhered to the Joanna Briggs Institute's methodology and was reported in accordance with PRISMA‐ScR guidelines. The search strategy considered key terms of ‘risk’, ‘clinical’ and ‘students’ in MEDLINE, Embase, CINAHL and grey literature. An inductive‐deductive approach was used for thematic data analysis.

**Results:**

We included 21 studies, which addressed risks in patient safety, student safety and incident response. Twelve studies reported on existing local practices, whereas nine evaluated new interventions. Ten studies intervened at the student level with education programmes, and 11 intervened at the faculty level with protocols and policies. Included studies involved students from medicine, nursing, dentistry, physiotherapy and psychology, with one study involving multiple disciplines.

**Conclusions:**

Risk governance in clinical education is an emerging field, with faculties at various stages of establishing a risk governance framework. Key requisites for risk governance are robust and streamlined processes for incident reporting, incident response and policy change. Further research is needed to understand the impact of centralised multidisciplinary governance.

**Abbreviations**
CESAR: Clinical Education Strategy and Risk
COVID‐19: coronavirus disease of 2019
MDHS: Medicine, Dentistry and Health Sciences

## 1 Background

Clinical education is an essential component in the training and accreditation of students in health professions [1]. In allowing students to observe and participate in patient interactions under supervision, clinical education bridges the gap between students' prior theoretical learning and future independent practice. It also immerses students in the institutional cultures of health services, preparing them for their future roles as members of a complex and dynamic workforce. By learning in clinical settings, students gain competence not only in discipline‐specific skills but also in communication with patients and colleagues alike.

However, clinical practice comes with inherent risk, which is the combination of an event's probability and its effect on objectives [2]. When inadequately managed, these risks can manifest as clinical incidents: unexpected, unintended events that may cause harm or disrupt care delivery [3]. Adverse events in healthcare have been found to be a major source of mortality and morbidity globally, imposing a great cost on both disability‐adjusted life years and healthcare resources [4]. With these events come opportunities for intervention; health services use risk management software that allows staff to report incidents and near misses, which then undergo root cause analysis and lead to interventions such as revised policies and guidelines [5]. As healthcare and treatment are almost always accompanied by risk, the aim of risk interventions is often to mitigate, but not eradicate, the risk [6].

Risk management in the context of clinical education for healthcare students has its unique complexities, not least due to the delicate balance between patient safety and student learning. Clinical education spans a wide range of settings, including hospital wards, outpatient clinics and primary care. Students' scopes of practice change as they progress through their degree. The types of incidents that dominate each healthcare discipline also vary. Needlestick injuries and biological exposure are shared across students in medicine, nursing and dentistry [7, 8, 9, 10, 11]. Medical students face mental health challenges [12], as do dentistry students [13], who also risk musculoskeletal injury [14]. Nursing students risk making medication errors [15], obtaining musculoskeletal injuries [16] and facing aggression from patients [17].

*Risk management in the context of clinical education for healthcare students has its unique complexities, not least due to the delicate balance between patient safety and student learning*.

The stakeholders at risk can be any combination of students, patients, the university and its partner health services in clinical education delivery. Where safety is concerned, incidents on clinical placement can also imply legal liability for the university [18]. Not only do these incidents impact the student's mental health and fitness to practise, but they also pose significant reputational and financial risks to the university. It is therefore imperative that the health faculties of universities have a process for capturing and responding to incidents occurring on clinical placement and use these to drive strategic programs that reduce the underlying risk. Although many studies have characterised the risks encountered by students on clinical placement and made recommendations for action [8, 12, 14, 15], there is yet to be a consensus on risk governance in clinical education.

In 2022, the University of Melbourne's Faculty of Medicine, Dentistry and Health Sciences (MDHS) established a new risk management initiative for exactly this purpose. The Clinical Education Strategy and Risk (CESAR) committee is a central portfolio that governs over risks and incidents arising from all healthcare disciplines within the MDHS faculty [19]. One of CESAR's objectives was to enable the sharing of insights across disciplines through centralised governance. Now in its third year, CESAR is set to undergo an evaluation of its processes and outcomes.

To our knowledge, there has yet to be a review of the risk governance landscape in healthcare students' clinical education. This scoping review aims to map current practices used by tertiary institutions to manage risks and incidents in clinical education for healthcare students. By synthesising the global context in clinical education risk governance, our findings will also contribute to the framework of CESAR's evaluation.

## 2 Materials and Methods

Since clinical risk governance, particularly in the context of healthcare students' clinical education, is still an emerging field [20, 21], a scoping review was considered most appropriate to map available evidence. To ensure a systematic and robust review of existing literature, this study was conducted in accordance with the Joanna Briggs Institute methodology for scoping reviews [22] and adhered to the Preferred Reporting Items for Systematic Reviews and Meta‐Analyses extension for Scoping Reviews (PRISMA‐ScR) guidelines [23]. The Population, Concept, Context (PCC) framework was used to define the review's scope and structure during the planning phase. The analysis framework combined an inductive‐deductive approach with the scoping review process outlined by Arksey and O'Malley and further refined by Levac and colleagues [24, 25, 26]. Ethics approval was not required as this study reviewed existing literature and did not conduct new research.

### 2.1 Search Strategy

The search strategy aimed to find both published and unpublished literature from all health disciplines. An initial limited search was conducted on MEDLINE (Ovid) to capture keywords contained in the titles and abstracts of relevant articles. A full search strategy was then developed for MEDLINE (Ovid) with the help of an experienced librarian (see Figure S1). It was translated for use with Embase (Ovid) and CINAHL (EBSCO) to ensure coverage of allied health disciplines. It focused on the intersection of three main concepts: healthcare students, risk management and clinical settings, as identified using a SPIDER table (Table 1). The final search was conducted on 4 March 2025.

**TABLE 1:** SPIDER table for conversion of research question into search strategy.

Research question	How are risks and incidents managed in the clinical education of healthcare students?
Sample	Healthcare students in clinical settings
Phenomenon of interest	Risks and incidents
Design	Observational or experimental
Evaluation	Interventions to manage risks
Research type	Reporting or evaluative

### 2.2 Eligibility Criteria

As this review is focused on risk governance involving students on clinical placement, any studies reporting on risk management for registered healthcare professionals were excluded. Studies with students making up part of their sample of healthcare workers were also excluded unless results pertaining to the student subset were explicitly separated. Included studies must concern management of risks or incidents related to having students on clinical placement; any studies concerning only academic performance, or only characterising but not managing clinical risks, were excluded. Similarly, commentaries and protocols were excluded on the basis of lacking a risk intervention. Given the rapidly changing technology and risk landscape, and to balance the changes brought by the COVID‐19 pandemic, we limited the publication year to exclude papers published before 2013. These criteria are summarised in Table 2.

**TABLE 2:** Eligibility criteria for student population in clinical context with risk intervention.

	Inclusion	Exclusion
Population	Healthcare students enrolled in a preregistration healthcare course	Registered healthcare professionals Healthcare students as a subset of the health workforce without explicit breakdown in results
Context	Clinical placement: clinical environment in which students learn, including primary, secondary and tertiary; public and private health services	Academic performance
Concept	Management of risks and incidents related to having students on clinical placement	Characterisation of a specific risk without intervention to manage the risk
Types of evidence	Primary empirical research Literature reviews Full‐text conference proceedings Full‐text articles	Commentaries Protocols
Publication year	From 2013 onwards	

### 2.3 Study Selection

Study selection was performed using Covidence by two independent reviewers (R.T. and J.M.S.). To test rigour in the eligibility criteria, the reviewers screened a random pilot sample of 50 articles using predefined eligibility criteria. The kappa statistic was calculated to assess interrater agreement for study inclusion, with a value of 0.80–0.90 (indicating strong agreement) being used as the predetermined threshold for acceptance [27]. The remaining titles and abstracts were then screened. Subsequently, full‐text articles of all relevant studies and potentially relevant studies from reference tracking were retrieved. Any disagreements that arose between the reviewers at each stage of the selection process were resolved through discussion, and if necessary, by a third reviewer (L.C.).

### 2.4 Data Extraction

Data extraction was performed independently by two reviewers (R.T. and J.M.S.) using a predefined extraction framework. Extracted information included the study objective, target population, study design, sample size, risk addressed, risk intervention and reported outcomes. Discrepancies between reviewers were resolved through discussion to ensure consistency and accuracy of extracted data. It was not necessary to contact the study authors for clarification or retrieval of missing information. The extraction template used is presented in Table 3; the full extraction table is included in Table S1.

**TABLE 3:** Extraction template.

Origin	Objective	Study design	Population	Sample size	Risk addressed	Intervention	Outcome

### 2.5 Data Analysis

Qualitative data extracted from included studies were analysed using thematic analysis, applying an inductive–deductive approach as described by Braun and Clarke [25]. Two reviewers (R.T. and J.M.S.) independently conducted open coding to enhance analytic rigour, with discrepancies resolved by consensus. Codes were iteratively grouped into themes through constant comparison, ensuring internal consistency and external distinctiveness. After discussion with supervisors (L.C. and L.N.), thematic synthesis focused specifically on strategies employed by tertiary institutions to manage risks and incidents during clinical placements of healthcare students. Identified themes were categorised across student and faculty‐level interventions, providing a structured synthesis of current risk mitigation practices within clinical education.

## 3 Results

### 3.1 Study Inclusion

**FIGURE 1:**
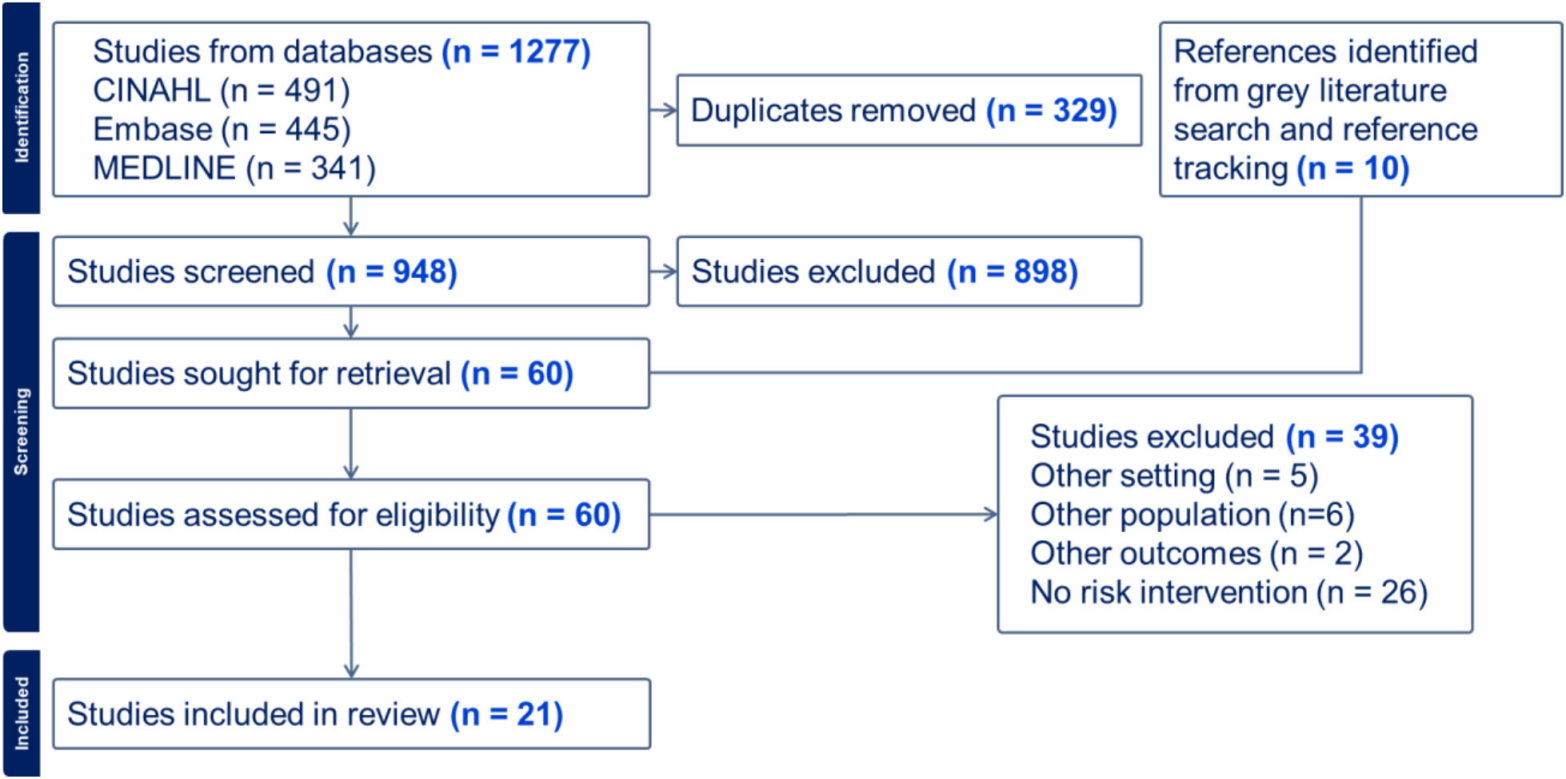
PRISMA flow diagram.

The search strategy retrieved a total of 1277 articles. After 329 duplicates were removed, 948 articles were screened based on title and abstracts, with 898 being excluded at this stage. Full‐text review was conducted for the remaining 50 articles, alongside 10 additional articles from grey literature and reference tracking. Of these, 39 articles were excluded. Reasons for exclusion included a lack of risk intervention (*n* = 26), other population (*n* = 6), other setting (*n* = 5) and other outcomes (*n* = 2). This yielded a total of 21 articles included in the review. The results of the search and study selection are presented in Figure 1.

### 3.2 Study Characteristics

The studies were categorised with respect to time of publication, country of origin, population and study design. All studies included in the review were full‐text journal articles in English published between 2013 and 2025. Among these, 20 were single‐nation studies, whereas one was multinational. Nine studies (43%) were conducted in the United States, with the remaining 12 being conducted in countries including South Korea, Australia, China, Canada, Iran, Saudi Arabia, Argentina, Brazil, Colombia, Ecuador, Spain and the United Kingdom. One study's risk intervention applied to both medicine and nursing, whereas all others focused on a single healthcare discipline, including nursing and midwifery (*n* = 11), medicine (*n* = 5), dentistry (*n* = 2), physiotherapy (*n* = 1) and psychology (*n* = 1).

### 3.3 Study Objectives and Outcomes

The risks addressed included patient safety risk due to student error (*n* = 5), student safety risk (*n* = 8) and more broadly placement incidents and response (*n* = 8). Of the 21 studies, 12 studies took a reporting approach, and nine were evaluative. The former described local risk governance practices in areas such as infection control and incident reporting; the latter assessed the efficacy of new risk interventions, which included gallery walks, gamified learning and online modules. Ten studies managed risks by intervening at the student level with tools such as education programmes, whereas 11 studies intervened at the faculty level by introducing protocols for reporting and responding to risk. Student‐level interventions' outcomes included self‐reported competence and assessed performance; faculty‐level interventions' outcomes encompassed incident counts and faculty feedback. These characteristics are summarised in Table 4.

**TABLE 4:** Characteristics of included studies.

Author year	Country	Study design	Discipline	Risk addressed	Level of intervention
Kitson‐Reynolds and Ferns, 2013	United Kingdom	Report	Midwifery	Incident response	Faculty
Disch and Barnsteiner, 2014	United States	Report	Nursing	Incident reporting	Faculty
Imperato et al., 2016	United States	Report	Medicine	Student safety	Faculty
Park et al., 2016	South Korea	Report	Medicine	Student safety	Faculty
Disch et al., 2017	United States	Report	Nursing	Incident response	Faculty
Emerson et al., 2018	United States	Report	Nursing	Incident reporting	Faculty
Franzblau and Haque, 2018	Canada	Evaluation	Medicine	Student safety	Student
Kim et al., 2018	South Korea	Evaluation	Nursing	Patient safety	Student
Graj et al., 2019	Australia	Evaluation	Psychology	Student safety	Student
Myers and Covington, 2019	United States	Report	Physiotherapy	Incident response	Faculty
Ryder et al., 2019	United States	Report	Medicine	Patient safety	Student
Yeh et al., 2019	United States	Evaluation	Nursing	Incident reporting	Student
Mills et al., 2020	United States	Evaluation	Dentistry	Student safety	Student
Chiou and Liu, 2021	Republic of China	Report	Nursing	Patient safety	Faculty
Ditton et al., 2023	Australia	Evaluation	Medicine	Student safety	Student
Lingawi et al., 2023	Saudi Arabia	Report	Dentistry	Student safety	Faculty
Padesh et al., 2023	Iran	Evaluation	Nursing	Patient safety	Student
Walker et al., 2023	United States	Report	Nursing	Incident reporting	Faculty
Gil‐Hernandez et al., 2024	Argentina, Brazil, Colombia, Ecuador, Spain	Report	Medicine and Nursing	Incident reporting	Faculty
Kim and Kim, 2024	South Korea	Evaluation	Nursing	Patient safety	Student
Zhao et al., 2025	People's Republic of China	Evaluation	Nursing	Student safety	Student

Incident reporting and response processes were improved in three main ways: proactively instigating incident reporting systems, implementing robust protocols for incident response and developing interventions to mitigate the risk of further similar incidents. For example, a nursing student's medication prescription error may be reported via an online form, triggering interviews with stakeholders involved, root cause analysis and ultimately review and revision of the nursing course's medication prescription curriculum [15].

A variety of student educational initiatives sought to address patient and student safety; formats included teaching with simulation, self‐learning and collaborative simulation. Program‐wide policies were employed to ensure student safety during outbreaks and whilst on overseas electives. Where risks overlapped across disciplines, management strategies also demonstrated similarities. These results are presented in Table 5.

*Where risks overlapped across disciplines, management strategies also demonstrated similarities*.

**TABLE 5:** Risks addressed and management interventions of included studies.

Risk addressed	Management strategy (discipline)	References
*Incident reporting and response* Faculty oversight of incidents Health provider awareness of incidents Student clinical performance	*Proactive* Incident reporting system (nursing, medicine) Just culture (nursing) *Reactive* Incident investigation and root cause analysis (nursing, midwifery, physiotherapy) Student support (midwifery) Communication with placement partners (nursing) *Intervention development* Targeted improvement of clinical education (nursing) Risk management education to students (nursing, medicine, psychology)	[3, 28, 29, 30] [31] [32, 33] [32] [29] [15, 34] [30, 35, 36, 37]
*Patient safety* Hazard perception	*Teaching and simulation* Risk identification education to students (nursing)	[38, 39, 40]
*Student safety* Physical injury Psychological harm Infection control Overseas elective	*Self‐learning* Photographic posture self‐assessment (dentistry) Psychological safety training (medicine) *Collaborative simulation* Violence assessment and de‐escalation training (nursing) Small‐group ethical dilemma training (medicine) *Program policy* Social distancing, altered placement attendance (medicine, dentistry) Motivation screening, vaccination, limits to scope of practice (medicine)	[41] [42] [43] [44] [45, 46] [47]

## 4 Discussion

Our global health system's vulnerability to risk was exposed in the COVID pandemic in 2019. In particular, healthcare worker shortages highlighted the importance of a sustained supply of competent healthcare students for the workforce [48]. Although there is an abundance of literature that characterise the risks related to clinical placement [11, 49], this is the first review to our knowledge to comprehensively map the interventions used by tertiary healthcare programmes to manage such risks on clinical placement. We will discuss the review findings with respect to the type of risk being governed, the health discipline implicated and the method of intervention.

Existing evidence in the field of clinical education risk governance can be classified into two categories: incident reporting and response, and specific risk intervention.

### 4.1 Incident Reporting and Response

This review confirmed that risk governance in clinical education is an emerging field, with most faculties lacking a formal reporting system for incidents that occur on clinical placement [50].

#### 4.1.1 Proactive Processes

Of the health disciplines, nursing seems to be the forerunner, with Chiou and colleagues [3] developing the first incident reporting system for students for a nursing school in Taiwan. Although the system drew inspiration from its precursor used by hospital staff, its establishment recognised two key differences driving the need for a student incident reporting system: that students represented a population with risks distinct from staff, and that universities providing clinical education must have oversight of the risks associated with such education.

In the United States, an online incident reporting form was similarly designed for nursing clinical education [28]. To encourage engagement with incident reporting, Disch and Barnsteiner advocated for a shift towards ‘just culture’, focused not on attributing individual blame for incidents but on improving the system [31]. The practice of incident reporting was further popularised in the years that followed. Incident findings were converted into actionable feedback for curriculum design [34]; the reporting tool was improved via consultation with the legal department, its outreach was increased via promotion to staff and students, and the feedback loop was closed by sharing reports with the nursing school's hospital partners [29].

#### 4.1.2 Reactive Processes

Parallel to the collection of incident reports is the faculties' response to each report. One study described a model that included an independent investigation of the reported incident, followed by a 2‐h meeting between the academic lead and the involved nursing student for incident debrief and generation of a written statement as evidence. A further meeting was offered after the student's return to placement as an opportunity for support [32]. A physiotherapy programme also used a three‐step framework to approach student failure in clinical education. They examined student factors contributing to the incident, analysed how the situation was influenced by the clinical environment and evaluated the above factors for predictability and modifiability in forming an action plan [33]. A multifaceted and supportive incident response process emerged from these studies.

Parallel developments in reactive and proactive branches of incident reporting set the stage for upscaling the incident reporting process. An incident reporting system was developed for medical and nursing students in Argentina, Brazil, Colombia, Ecuador and Spain to permit the sharing of incident learnings across institutions and disciplines. It achieved a secondary benefit of training students in performing root cause analysis for incidents [30].

#### 4.1.3 Intervention Development

In addition to contemporaneous risk mitigation, incident reporting systems also enable longitudinal surveillance. A decade after establishing their reporting system, Chiou and Liu [15] noted the recurring theme of medication errors among incidents reported by nursing students and designed a project‐based learning curriculum aimed at improving drug calculation and medication knowledge. This reduced category B medication errors from 74 incidences in 3 years pre‐implementation to 53 incidences in 3 years post‐implementation.

Such improvements deriving from incident reporting trends hinge on students' consistent use of the incident reporting system. A study on medical students addressed this by implementing and evaluating a patient safety reporting curriculum. It comprised a structured incident analysis report book‐ended by two interactive case‐based sessions and was found to improve medical students' self‐reported comfort with disclosure of medical errors [35]. Another study found that simulation experience in reporting an incident improved medical students' reporting performance and confidence [36]. Similarly, an online simulation‐based learning programme on risk awareness enhanced psychology students' recognition and mitigation of risk [37].

Proactive implementation of an incident reporting system, reactive incident analysis and intervention development were key components for a faculty‐level approach to incident reporting and response.

### 4.2 Risk Interventions

Apart from incident reporting and risk awareness measures to manage risks collectively, the review also uncovered interventions intended to mitigate risks in the following areas.

#### 4.2.1 Patient Safety

Three studies on nursing students evaluated patient safety risk interventions. A hazard perception training programme challenged students to identify hazards in clinical situation pictures, consequently improving students' hazard sensitivity to falls, medication, procedures and preoperative time out [38]. Three 3‐h didactic sessions of clinical risk management training increased students' patient safety competency and comfort in speaking up by covering types, identification and reporting of risks to patient safety [39]. A more extensive 5‐day patient safety incident disclosure programme similarly enhanced students' self‐efficacy and knowledge in patient safety and incident disclosure [40].

#### 4.2.2 Student Safety

Student safety was implicated both physically and psychologically. In the physical aspect, a weekly photographic self‐assessment protocol improved dental students' posture over time according to the Rapid Upper Limb Assessment, thereby reducing the risk of musculoskeletal injury [41]. Nursing students' risk of injury from patient aggression in the psychiatric setting was mitigated through improved violence risk assessment ability after an interactive game simulation covering risk factors, previolence indicators, verbal de‐escalation and teamwork during violence incidents [43].

With regard to psychological safety, medical students reported learning new ways to approach dilemmas on clinical placement after engaging in a gallery walk, where they rotated through confronting scenarios with small‐group discussion following each case [44]. Another evaluative study focused on cultivating psychological flexibility in medical students using a mobile app with self‐learning modules based on Acceptance Commitment Therapy, which reduced self‐reported psychological distress but did not change exhaustion levels [42].

#### 4.2.3 Infection Control

Two reports described institutional measures taken to mitigate infection risk during outbreaks. A South Korean medical school maintained zero infections despite continued teaching during a Middle Eastern Respiratory Syndrome outbreak by pausing placement, providing personal protective equipment during practical classes and transitioning to remote delivery [45]. Meanwhile, a dental clinic in Saudi Arabia was able to preserve placement provision during COVID‐19 with extended clinic hours, alternating student attendance and strict social distancing and cleaning protocols [46].

#### 4.2.4 Overseas Electives

One medical school reported on their practices to ensure the safety and preparedness of medical students participating in their overseas electives. Students were screened for motivation, academic record and interpersonal skills; they were offered relevant vaccinations, banned from performing invasive procedures and provided opportunities for debrief upon their return. Such measures kept the adverse event count to 26 incidents over 36 years of the programme. These included allergic reactions, gastrointestinal infections, robbery, scams, animal bites, inability to adjust and sexual and racial harassment [47].

Interventions to improve risks in patient and student safety on clinical placement were mostly educational, although they varied in didactic, collaborative or remote delivery. Infection control and safety on overseas electives were achieved using robust protocols and strict implementation.

### 4.3 Knowledge Gaps

This review has identified several key knowledge gaps. Although we found risk interventions in medicine, nursing, dentistry, physiotherapy and psychology, most of the literature surrounded medicine and nursing risks, with a relative lack of evidence in other disciplines.

In addition, there was a gap in how faculties respond to incidents where significant harm is done to patients or students. Although lower impact incidents such as needlestick injuries are more prevalent and likely to predominate the incident reporting scene, it is crucial that faculties have a protocol in the event of a high‐risk incident that puts patient safety, student well‐being or the university's reputation under serious threat.

Finally, the costs and benefits to a multidisciplinary governance framework as opposed to a single discipline approach are yet to be seen, with only one of 21 included studies applying an intervention to both medicine and nursing [30]. However, where risks overlapped across disciplines, interventions also demonstrated similarities, providing some justification for insight sharing through centralised governance.

### 4.4 Strengths and Limitations

To date, this review is the first to map risk interventions in healthcare students' clinical education, providing a valuable synthesis of existing evidence. All included studies were peer‐reviewed journal articles, which may indicate greater reliability. Both reviewers were final‐year medical students with experience representing students on clinical education committees. R.T. has been a representative on CESAR since its 2022 instigation; J.M.S. was in the Student Placement Advisory Group. This insider perspective may have contributed positively to the review's rigour.

Although every effort was made to conduct a comprehensive search, it is possible that relevant studies were missed. The publication year was restricted to ensure currency of risk governance practices but may have caused earlier important works to be overlooked. Studies that sampled both registered health professionals and students were excluded to maintain the focus on students, which may have reduced the breadth of risk interventions discussed. As the included studies originate from different nations, the risk interventions may not produce comparable effects when replicated in a different setting due to the contextual specificities of risk perception.

### 4.5 Implications

The findings presented in this review offer important insights for practice. A robust incident reporting and response protocol is essential to the safe and sustainable provision of clinical education for healthcare students. It provides valuable opportunities for intervention to target recurring incidents. Risk interventions reported in this review also serve as a reference for programmes seeking to improve their own risk management practices.

The types of risks addressed in this review overlapped with what had been reported to CESAR. No other centralised, multidisciplinary risk governance portfolio like CESAR has been noted in the existing literature. It remains to be seen whether this governance structure proves superior to the single discipline approach.

As the healthcare landscape is transformed by new technologies, so too will its associated risk governance. Healthcare education providers should endeavour to plan ahead in anticipation of such changes: what new risks might artificial intelligence bring to clinical education, and how could it be used to mitigate existing risks? Rather than reactive risk interventions, proactive governance will better prepare us for change.

## 5 Conclusion

This scoping review suggests that risk governance in clinical education is an emerging field, with health faculties at various stages of establishing a risk management framework. We found the key requisites for risk governance to be robust and streamlined processes for incident reporting, incident response and policy change. We also discovered valuable insights in managing specific risks pertaining to patient and student safety, from which inspiration can be drawn to inform our own practices. Saliently, there appears to be a gap in the literature for centralised governance and sharing of risk governance practices across disciplines. As the clinical education landscape continues to evolve, so too must risk interventions to ensure safe and sustainable provision of clinical education for healthcare students such that they become competent health practitioners of the future.

*This scoping review suggests that risk governance in clinical education is an emerging field, with health faculties at various stages of establishing a risk management framework*.

## Author Contributions

**Raelynn R. Tong:** conceptualization, investigation, writing – initial draft, writing – review and editing. **Je Min Suh:** investigation, writing – review and editing. **Lisa Cheshire:** conceptualization, supervision, and writing – review and editing. **Lucio Naccarella:** conceptualization, supervision, and writing – review and editing.

## Funding

The authors have nothing to report.

## Ethics Statement

The authors have nothing to report.

## Conflicts of Interest

The authors declare no conflicts of interest.

## Supporting information


**Figure S1:** MEDLINE (Ovid) Search strategy.
**Table S1:** Extraction of included studies.

## Data Availability

Data sharing is not applicable to this article as no datasets were generated or analysed during the current study.
