# Supplementary material for: Risk Governance in Clinical Education for Healthcare Students: A Scoping Review
Source: Clin Teach. 2026 Feb 3;23(2):e70355. doi: 10.1111/tct.70355 (PMC12865877; doi:10.1111/tct.70355)
Supplement: Supplementary file 1 — Figure S1: MEDLINE (Ovid) Search strategy. Table S1: Extraction of included studies. [file TCT-23-e70355-s001.docx]

**Risk governance in clinical education for healthcare students: a scoping review**

**Appendix**


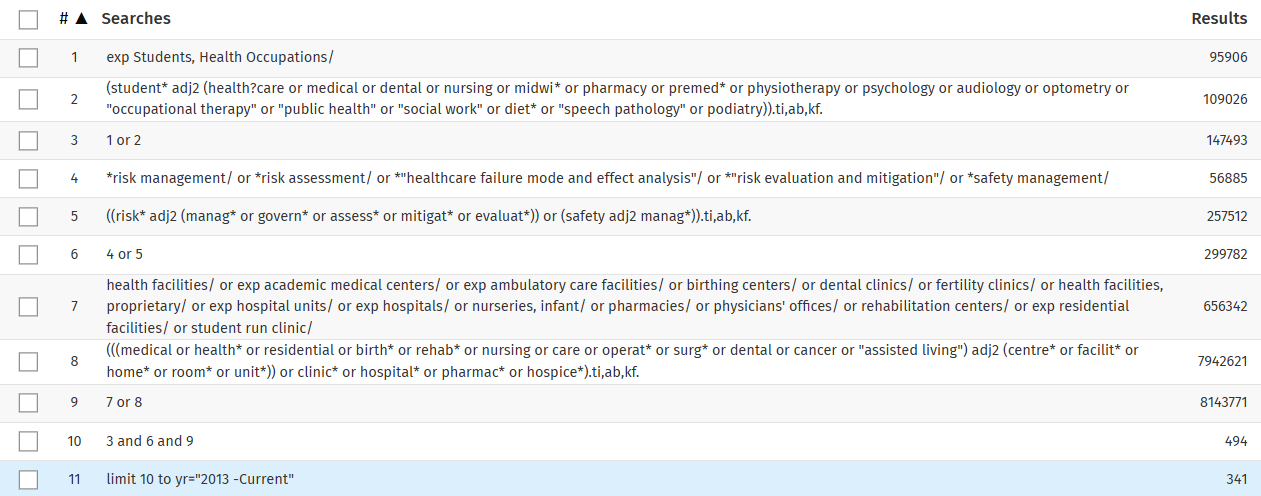


Figure 1. MEDLINE (Ovid) Search strategy

Table 1. Extraction of included studies

| **Author Year** | **Origin** | **Objective** | **Study design** | **Population** | **Sample size** | **Risk addressed** | **Intervention** | **Outcome** |
| --- | --- | --- | --- | --- | --- | --- | --- | --- |
| Kitson-Reynolds and Ferns, 2013 | UK | To share the model used to address and support student midwives through clinical incidences. | Report | Midwifery students | N/A | Incident response | Academic lead of student incident management trained in expert witness program. When student asked to provide evidence towards an investigation, an independent investigation should be conducted by the supervising midwife alongside a risk analysis and root cause analysis. Then report to lead for student incident mangement. 2 hour meeting between academic lead and student with breaks. Read aloud statement without interruption, then debrief, then read with interruptions, then academic lead constructs a draft statement and sent to legal services department of university. Meetings with the Trust are attended by the academic lead. Another meeting after return to placement for reflection. Support by supervisor in first shift back. | Less requirement for further enquiry as quality of student statement is high. |
| Disch and Barnsteiner, 2014 | USA | To discuss the issues and challenges in creating and piloting an online occurrence reporting tool for errors and near misses by prelicensure nursing students and to provide an occurrence reporting tool that can be used by schools of nursing. | Report | Nursing schools | 10 | Incident reporting | Seminar with head of school to introduce and gain prelimary feedback. Four additional seminars for other faculty to educate on electronic tool and reporting process. Tool collected student demographic, school characteristic, nature of error/near miss, whether it was reported, consequences for student/staff/patient, originator of report. | Positive feedback, easy to use. However need cultural change and centralised national data repository for benchmark data, curricular improvement and peer pressure. |
| Imperato et al., 2016 | USA | To share the model developed to ensure health, safety and preparedness of students participating in global health elective. | Report | Fourth year medical students | 386 | Overseas elective: infection, legal, health insurance and medical indemnity. | Screening for motivation and commitment. Academic record and interpersonal difficulty in third year. Interview: ability, interpersonal skills, non-English language skills if applicable. Site selection of health service and housing. Ban of invasive and surgical procedures. Agreement and Release form to waive university of liabilities. Advice from graduates to prospective students. Evaluation of safety, security, personal health, performance and paper submission. Debrief post experience and medical referral when necessary. | 26 adverse events over 36 years: infection, physical assault/robbery, scam, sexual and racist harassment, animal bite. |
| Park et al., 2016 | South Korea | To share risk management experience in MERS outbreak | Report | Medical students in Sungkyunkwan SOM | 125 | Infection | Emergency committee with aims of safety, learning and anxiety. Discontinued placement but continued hospital teaching with hand hygiene, mask use and temperature monitoring. Discontinued hospital teaching and shifted summer break to buy time. Relocated teaching and switched to remote. Communication between medical school and hospitals. | None infected |
| Disch et al., 2017 | USA | To determine whether prelicensure nursing programs have a policy for reporting and following up on student clinical errors and near misses, a tool for such reporting, identification of trends and follow-up with stakeholders. | Report | Nursing schools | 494 | Students' errors and near misses | Survey about differentiation between errors and near misses, existence of written policies or tools for handling such events, process for identifying trends, process for followup with stakeholders. | 55% no reporting tool, 50% no follow up policy, 81% no trend tracking tool. |
| Emerson et al., 2018 | USA | To describe experience of clinical faculty with nursing students' clinical errors and near misses to create a reporting form. | Report | Part time clinical nursing faculty | 28 | Incident reporting and risk management | Quality and Safety Education for Nurses for part time clinical faculty. Clinical error and near miss reporting form. | Shared findings across faculty, emphasis on bedside assessment, longer lab time, hiring educators from practice environments. |
| Franzblau and Haque, 2018 | Canada | To share the experience of using a gallery walk activity to facilitate students with tools and confidence needed to speak up during future encounters. | Evaluation | Medical students | 164 | Speaking up | Teams of three or four students rotated around five poster stations that displayed representative cases derived from prior clinical students' experiences (senior doctors exhibiting unprofessional behaviour). At each station, teams discussed the case, generated suggestions for how they might respond and wrote ideas on the poster. Then all participated in a 25 minute large group discussion of all generated susggestions to identify feasible responses. | 62% learned new ways to approach ethical dilemmas, 79% could see themselves using these strategies in the future and 57% would recommend this session to future classes. |
| Kim et al., 2018 | Korea | To develop a safety sensitivity training program for patient safety as a new education course for nursing students and also identify the effects of training on risk sensitivity and confidence in safety control after utilising the program. | Evaluation | Final year nursing students | 107 | Patient safety | Three hour hazard perception training for 7 to 9 participants at a time. Student presented with situation picture and asked to report hazards, discussion about preventative actions for each found hazard. Five situation pictures in total. | Compared to the control group, the experimental group's hazard sensitivity improved for falls, preoperative time out, invasive treatment, and medication. No change found for telephone medication orders. |
| Graj et al., 2019 | Australia | To evaluate the impact of Risk Aware, an online blended simulation-based learning program, upon student preparedness and confidence for clinical placement. | Evaluation | First year psychology students | 139 | Risk on placement for students | Seven modules addressing self-management, interpersonal risk, infection control, aggression and physical violence, environmental risk, psychological and emotional risk and education-specific risks. | Regarding clinical placement risk: increased knowledge, increased confidence. |
| Myers and Covington, 2019 | USA | To share experience of developing a mechanism for formal, objective analysis of student failure in the department of physiotherapy clinical education experiences. | Report | Physiotherapy department | N/A | Response to student failure | Analysis of Clinical Education Situations 3-step framework: examination of central situation and student factors, analyse how situation was influenced by clinical env/academic curriculum and director, evaluate factors for impact, predictability and modifiability. | Formalised mechanism to evaluate student failure situations through multifaceted approach accounting for all stakeholders, resulting in greater collaborative effort between academic and clinical faculty. |
| Ryder et al, 2019 | USA | To describe the development, implementation and evaluation of the patient safety reporting curriculum. | Report | Third-year medical students | 131 and 16 control pre-curriculum | Incident reporting for patient safety error | Structured, focused written report for students to analyse a medical error they witnessed. Bookended by 2 interactive case-based sessions led by faculty. Aims of improving understanding of how and why errors occur, comfort reporting, emotional effects. | Self-reported improved attitudes and increased comfort with analysis and disclosure of medical errors. Four systemic changes: isolation of flu patients, standardised precautions against self-harm in patients. |
| Yeh et al., 2019 | USA | To test the conceptual model of deliberate practice within the theoretical framework of mastery learning in providing online simulation-based opportunities for prelicensure nursing students to practise reporting patient critical incidents. | Evaluation | Third-year nursing students | 43 | Incident reporting | Professionally recorded audio stories, narrated with dialogue and though processes from multiple perspectives, supplemented by fictional patient information. Then students required to report the incident to another provider. 2 sessions completed. | Intervention group showed greater incident reporting performance improvement, confidence improvement than the control group. |
| Mills et al., 2020 | USA | To determine if dental hygiene students' self-assessment using the Rapid Upper Limb Assessment (RULA) tool reduced the risk of MSD development and explore students' experiences using a risk tool. | Evaluation | Dental hygiene students | 21 | Musculoskeletal disorder | Students photographed over 4 weeks during active clinical patient care, from front and profile views. After week 1, 30 minute workshop to teach RULA using an example photograph. Given access to that week's photographs for self-assessment. Weekly reviews continued. Then focus group to discuss experience. | Significant improvement on average RULA scores week to week. Qualitatively: increased self-awareness. |
| Chiou and Liu, 2021 | Taiwan | To analyse the causes and patterns of medication errors events based on the incident reporting system, and to develop teaching strategies. | Report | Nursing students | 31 | Medication error | Project based learning aimed at improving drug calculation and medication knowledge. | Category B medication errors decreased from 74 in 3 years to 53 in 3 years after implementation of PBL |
| Ditton et al., 2023 | Australia | To evaluate the effectiveness of an app-delivered ACT inrevention for medical students with respect to burnout, wellbeing and psychological flexibility and inflexibility outcomes; secondary psychological outcomes. | Evaluation | First, second, fourth and fifth year medical students | 108 | Psychological burnout | ACT-based psychological flexibility training program via app, developed by clinical psychologists. Learn the concepts then learn the behaviours. Processes include acceptance, committed action, defusion, present-moment awareness, values and self-as-context. Choose one of six as most relevant for the day, activity recommended was individualised or not. | Self-report psychological outcome: exhaustion (same), hedonic and eudaimonic (improved in nonindividualised), psychological flexibility (improved), psychological distress (improved stress). |
| Lingawi et al., 2023 | Saudi Arabia | To share experience of development and application of a modified infection control protocal at dental teaching hospital during second wave of COVID-19 pandemic. To evaluate the impact of implemented strategies on preparations for future requirements in clinical dental education. | Report | Dental students | N/A | Infection | Infection control committee established new policies, on which dental students were given orientation and mandatory training lectures. Training on PPE donning and doffing. Cleaners trained in environmental cleaning and disinfection. Advised to download government app for contact tracing. Triage station for visual and temperature screening and triage form at hospital entrance. Separate entry and exit pathways for patients and students. Marked seating for social distancing. Hand sanitising stations. COVID-19 contact tracing committee to receive reports from attendees of hospital. Every second dental pod was left vacant, students divided into two groups to alternate in placement attendance, clinic schedule extended from 8 to 12 hours (0800-2000), fallow time in between for disinfection of the unit. Minimised aerosol generating procedures by using hand instrumentation and minimally invasive techniques where possible. Five uses of each N95 respirator by same HCW according to policy. Strict implementation of infection control policies and any violations were reported for action. | 4.5% of staff and students tested positive: 18/396 cases. This is in the setting of 1272 cases in Makkah, a COVID hotspot in Saudi Arabia. Reduced patients treated during second wave compared to pre-COVID. |
| Padesh et al., 2023 | Iran | To determine the effect of clinical risk management training on patient safety competency, attitudes and comfort in speaking up. | Evaluation | Third and fourth year nursing students | 56 | Errors in patient care | Clinical risk management training in 3x3h sessions. Combination of lectures, film screenings and group discussions. Teaching on types of risk and errors and risk identification and reporting. | Increased patient safety competency and comfort in speaking up. |
| Walker et al., 2023 | USA | To share the experience of developing and implementing a successful error and near misses reporting process as a component of just culture across two different schools of nursing. | Report | Nursing programs | 2 schools with enrollment of 450 each | Incident reporting | Desire, development, education and engagement and feedback, and analysis. Started with quality and safety committee to analyse errors. Then used student input and hospital tool as template to set up quality and safety reporting tool for students. Reviewed by legal department and put into use. Drop boxes placed in nuring lab, office and library. Awareness and stakeholder engagement (LMS reminders, student association meetings, faculty meetings, video at student orientation, student newsletter, faculty certificate if student fills a report, student certificate and badge for logging a "good catch"). Committee reviewed reports and made recommendations. Report learning shared with other students by encouraging narrative writing by reporting student in the newsletter. Committee shared reports with hospital partners. | N/A |
| Gil-Hernandez et al., 2024 | Argentina, Brazil, Colombia, Ecuador, Spain | To develop a patient safety incident reporting system tailored to the needs of healthcare discipline students. To study the performance of different groups of students in the use of the platform and train them on the correct procedures for reporting. | Report | Medical and nursing students in clinical years | 105 students in clinical years of medicine, nursing, and medical specialisation programs, 147 reports | Incident reporting | Used Spanish database of safety incidents to develop codified reporting form. Education seminar on reporting and patient safety with academic and financial incentives. Assessment of reports and feedback on experience based on quality and completeness of information provided, adequate risk analysis and realistic prevention plan. | Root cause analysis strong, but improvement plan weak. |
| Kim and Kim, 2024 | South Korea | To investigate the effect of a patient safety incident disclosure education program on the knowledge and perception of disclosure, attitudes toward patient safety and disclosure self-efficacy of fourth-year nursing students. | Evaluation | Fourth year nursing students | 55 experimental, 114 control | Patient safety incident disclosure | 6x 4-5p group programs, each for 5 days. Modules covered orientation, disclosure, obstacles to disclosure, methods, examples, guidelines, activation strategies and case studies. | Increased patient safety incident disclosure knowledge, perception and self-efficacy. No difference in attitude towards patient safety. |
| Zhao et al., 2025 | China | To measure the effect of integrating theme game-based learning into psychiatric violence risk assessment and response training on nursing student psychiatric violence-related knowledge, skills and self-confidence. | Evaluation | Full time third-year nursing students with existing training in basic psychiatric nursing | 104 | Student exposure to violence in psychiatric nursing | Game with six sub-themes: identifying psychiatric workplace risk factors, assessing supplies sent by family, identifying pre-violence indicators in psychiatric patients, labour division during violence incidents, verbal de-escalation techniques, protective restraints. Control group underwent simulation training. | Intervention group scored higher in violence risk assessment ability. Similar scores in total score and violent incident reporting and mitigation. |
